# A comparative molecular docking study of curcumin and methotrexate to dihydrofolate reductase

**DOI:** 10.6026/97320630013063

**Published:** 2017-03-31

**Authors:** Yahya Hobani, Ahmed Jerah, Anil Bidwai

**Affiliations:** 1 College of Applied Medical Sciences Jazan University, Jazan, Kingdom of Saudi Arabia;; 2 Index Medical College Hospital & Research Centre, Indore, Madhya Pradesh, India;

**Keywords:** curcumin, methotrexate, docking, AutoDock, DHFR, drug design

## Abstract

Interaction of curcumin (CUR) with the enzyme dihydrofolate reductase (DHFR) was studied by molecular docking using AutoDock 4.2 as the docking software application. AutoDock 4.2 software serves as a valid and acceptable docking application to study the interactions of small compounds with proteins. Interactions of curcumin with DHFR were compared to those of methotrexate (MTX), a known inhibitor of the enzyme. The calculated free energy of binding (ΔG binding) shows that curcumin (ΔG = -9.02 kcal/mol; Ki = 243 nM) binds with affinity comparable to or better than MTX (ΔG = -8.78 kcal/mol; Ki = 363 nM). Binding interactions of curcumin with active site residues of the enzyme are also predicted. Curcumin appears to bind in a bent conformation making extensive VDW contacts in the active site of the enzyme. Hydrogen bonding and pi-pi interaction with key active site residues are also observed. Thus, curcumin can be considered as a good lead compound in the development of new inhibitors of DHFR, which is a potential target of anti-cancer drugs. The results of these studies can serve as a starting point for further computational and experimental studies.

## Background

Turmeric (from Curcuma longa) is a yellow colored spice extensively used in daily food in Asia, particularly in India. It has been used in traditional medicine since ancient times. Turmeric has been a subject of extensive research for many years and its therapeutic potential against several diseases including cancer, CVD, lung and liver diseases etc. has been studied. It is shown to have some preventive as well as therapeutic effect in diseases without causing any toxicity [1-3].

The major active component present in turmeric is curcumin (CUR). Numerous lines of evidence indicate that curcumin possesses anti-inflammatory [4-6], hypoglycemic [7,8], antioxidant [9], wound healing [10], and anti-microbial activities [11]. Many clinical trials using curcumin as a therapeutic agent are under way [12]. Curcumin can bind to a number of target molecules to modulate their biological activity. In some instances, such binding has been studied using computational methods like molecular docking. With many target proteins, curcumin has shown strong binding affinity with a binding constant in the nanomolar to micromolar range. Chemically, curcumin is diferuloyl methane and its systematic chemical name is 1,7-bis (4- hydroxy-3-methoxyphenol)-1,6-heptadiene-3, 5-dione. In the structure a methylene bridge links two ferulic acid residues. The overall structure comprises two hydrophobic phenyl domains connected by a flexible linker. Curcumin can exist in many different conformations and this adaptability confers curcumin with the ability to bind directly to various protein targets. The conformational diversity allows for maximizing hydrophobic contacts with the protein to which it is bound. Although curcumin is generally hydrophobic, it has phenolic and carbonyl functionalities on the ends and in the center of the molecule that can be involved in hydrogen bonding with a target macromolecule. Curcumin also exhibits keto-enol tautomerism due to its b-diketone moiety and in solution and solid phase, it can exist entirely in the enol form [13,14]. In the enol form, the midsection of the molecule can serve as both donor and acceptor in hydrogen bonds. Positively charged metal ions found in target proteins are chelated in enol form [15]. Hence, the many possible mechanisms with which curcumin can interact with targets are a result of the availability of many modes of interaction including hydrophobic, p–p, H bonding, metal chelation etc.

Dihydrofolate reductase (DHFR) is an enzyme crucial for cell proliferation and cell growth. It uses NADPH as electron donor to reduce dihydrofolate to tetrahydrofolate. Tetrahydrofolate and its derivatives serve as 1-C donors in purine synthesis and thereby nucleic acid synthesis essential for cell proliferation and cell growth [16]. DHFR has been a target for anti cancer drugs since long and several therapeutic agents have been developed to target this key enzyme [17,18]. FDA has approved some of these for use while some are still in clinical trials. For cancer chemotherapy DHFR still remains an attractive target. The major drugs that have been developed to target DHFR are the folate analogs such as methotrexate (4- amino-10-methylfolic acid) and aminopterin (4-aminofolic acid). Being structural analogs of the substrate folate, these drugs competitively inhibit the enzyme. In methotrexate, an amino group replaces the 4-hydroxyl group of folate [16,19-23]. The active site of DHFR comprises the amino acid residues Ile-7, Leu-22, Phe-31, Phe-34, Arg-70, Val-115 and Tyr121. The NMR structure of DHFR shows an eight-stranded β- pleated sheet in the center of the molecule. Of the eight strands seven are parallel and the eighth runs antiparallel. Successive β strands are connected by four α helices [19,22]. A major sub domain surrounding the active site contains a loop of residues 9- 24 called “loop 1” (also termed “Met20 loop”). A conserved Pro- Trp dipeptide, of which the tryptophan is involved in substrate binding, is found towards the N-terminal of the structure [21]. The Met20 and other loops near the active site are highly flexible and promoting the release of the tetrahydrofolate product after the catalytic reduction of dihydrofolate by NADPH [16]. The nicotinamide ring of the NADPH is stabilized by the Met20 loop which promotes the transfer of hydride from NADPH to dihydrofolate [19,24].

Computational methods such as molecular docking are very useful and reasonably reliable for prediction of putative binding modes and affinities of ligands for macromolecules. Such methods are gaining popularity because the experimental determination of complex structures is rather difficult and expensive. Over the years, the speed and accuracy of computational docking methods has improved and these methods now play an important role in structure-based drug design [25-31].

The present study incorporates results of molecular docking of curcumin with the monomeric A subunit of DHFR. The binding is compared to the binding of MTX a known inhibitors of the enzyme. The A subunit of DHFR is referred to as DHFRA.

## Methodology

Version 4.2 of the molecular docking software AutoDockR [32], obtained from The Scripps Research Institutes, San Diego, CA, USA, was used in this study. AutoDock Tools [ADTR] [32,33] obtained from the same source was used as the GUI for AutoDockR 4.2 and for preparation of the protein and ligand for docking.

### Preparation of protein and ligand

The three dimensional structures of DHFRA and MTX were obtained from the PDB file 1DRE. The structural coordinates of CUR (ID: ACD0022) were obtained from the database of anticancer molecules, ACD. For docking experiments, the protein and the ligands were prepared using ADTR. Gestgeiger partial charges were assigned after merging nonpolar hydrogens. Torsions were applied to the ligand by rotating all rotatable bonds. Protein was kept rigid. Both the protein and the ligand coordinates were saved in the PDBQT format files which were used as input files for docking experiments in the next step.

### Docking

With AutoDockR 4.2, standard docking procedures for a rigid protein and a flexible ligand were used as per the user guide. A grid of 60x60x60 points in x, y, and z directions was built with a grid spacing of 0.375 Å using the AutoGrid component of the software. A distance dependent function of the dielectric constant was used for the calculation of the electrostatics map. Default settings were used for all other parameters. Lamarckian Genetic Algorithm [LGA] [34] was employed for docking simulations. LGA was implemented by creating an initial population of 150 individuals, applying random torsions to each of the 150 individuals, and performing a maximum of 2500000 energy evaluations in each docking run. At least 20 such runs were performed for both ligands. At the end of docking, the best binding modes were analyzed for various interactions using ADTR and RasMolR (Roger Sayle) [35] programs.

## Result and discussion

**Figure 1:**
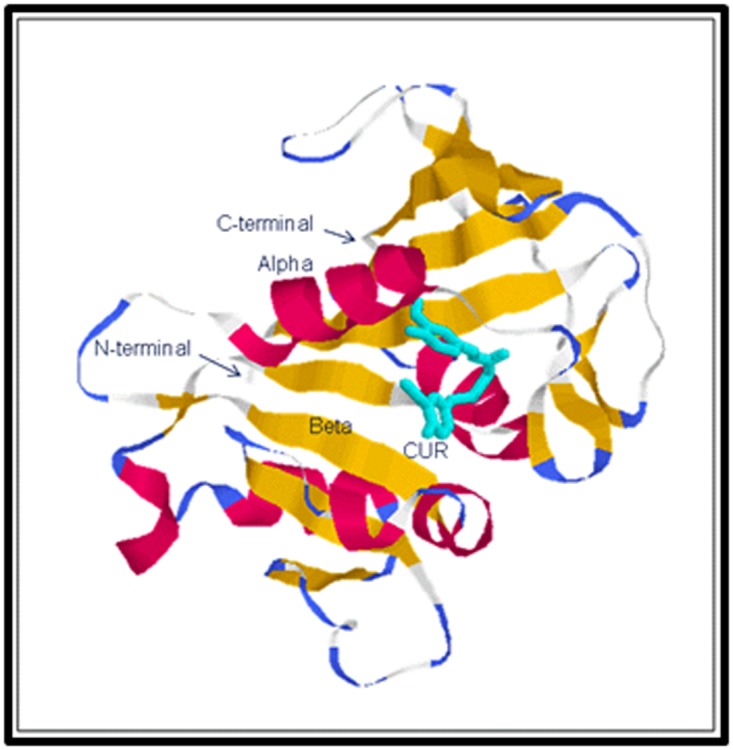
A structural rendering of the docked CUR-DHFRA complex showing CUR (cyan) in the active site of DHFRA. Secondary structural features of DHFRA are shown in standard color scheme (alpha – red, beta – yellow).

All the binding parameters of CUR and MTX obtained after docking with DHFRA are listed in Table 1. Estimates of total free energy of binding of the two inhibitors were -9.02 and -8.78 kcal/mol, respectively. The estimated KI values were 243 nM and 363 nM, respectively. The total free energy of binding (and hence the Ki) estimated for CUR is slightly lower than these values for MTX suggesting comparable binding of CUR with the enzyme. A structural rendering of the docked CUR-DHFRA complex, depicting docked CUR and secondary structural features of DHFRA, is shown in Figure 1.

Binding of folate and methotrexate to DHFR has been described in detail [36]. Several interactions of CUR with DHFRA are comparable to interactions of folate and MTX.

**Figure 2:**
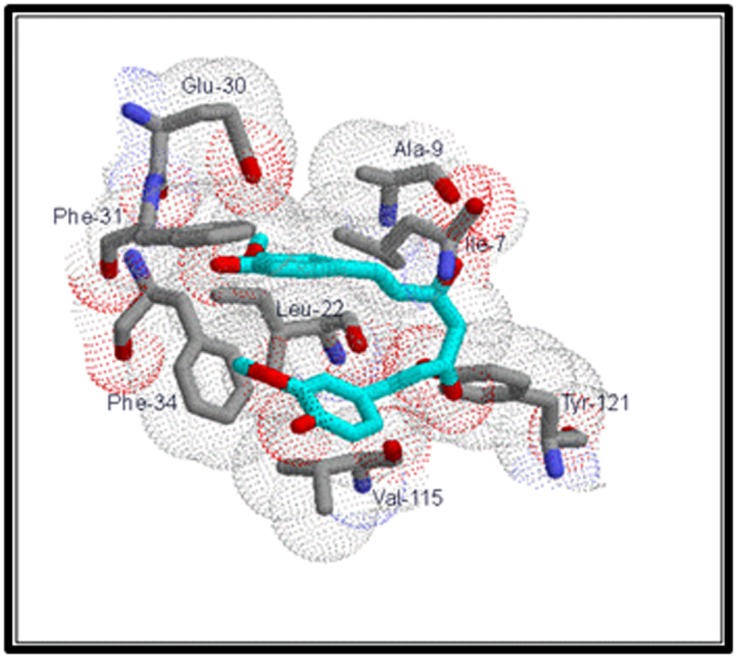
VDW interactions of CUR with active site residues of DHFRA. Active site residues are numbered as per the original PDB file, 1DRE. VDW radii are shown as dotted spheres. Active site residues are shown in CPK color scheme. CUR is shown in cyan (with all its oxygens in red).

**Figure 3:**
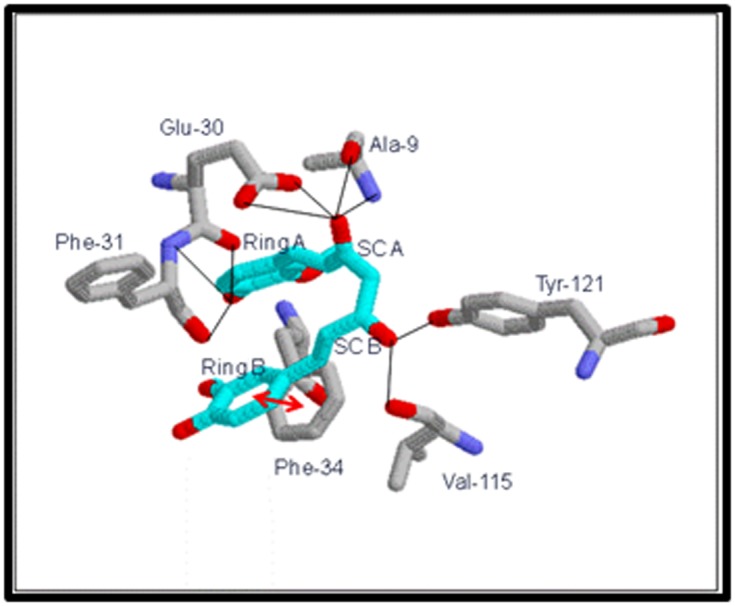
Significant interactions of CUR with the active site residues of DHFRA. Active site residues are numbered as per the original PDB file, 1DRE. Blue lines are hydrogen bonds and red double-headed arrows are pi-pi interaction. Residues are colored in CPK scheme. CUR is shown in cyan (with all its oxygen in red). SCA = side chain A and SCB = side chain B.

An analysis of the docked complex of CUR with DHFRA reveals several significant interactions of the ligand within the active site of DHFRA. Visual renderings of these interactions constructed in RasMolR are shown in Figures 2 and 3. The ligand CUR appears to bind in the active site in a bent conformation and makes extensive van der Waals contacts on either side with the active site residues of the enzyme (Figure 2). One phenyl ring, ring B of CUR, is in pi-pi stacking interaction with the phenyl ring of Phe- 34 in the active site pocket. In folate and MTX binding also hydrophobic contacts are made with the bulky side chains of Phe-31 and Phe-34, which cover one face of the pteridine rings. Nonpolar interactions occur with the side chains of Ile-7, Ala-9 and Val-115 and with some main chain atoms of Val-8 and Ala-9 in folate and MTX binding similar to interactions of CUR with Ile-7, Ala-9, Leu-22 and Val-115. Several hydrogen-bonding interactions are observed between active site residues and the phenolic and side chain OH groups of CUR (Figure 3). The OH of side chain A in CUR H bonds with (i) main chain NH and CO of Ala-9 and (ii) carboxyl O1 and O2 of Glu-30. The A ring phenolic OH in CUR H bonds with (i) main chain CO of Glu-30 and (ii) main chain CO and NH of Phe-31. Notable among these are the H bonds with Glu-30, which are also seen in folate and MTX binding. Glu-30 carboxylate makes H bonds with 2-amino and N3 in folate and 2-amino and N1 in MTX. Additionally in MTX binding, H bonds are observed between 4-amino of MTX and the main-chain carbonyls of Ile-7 and Val-115 [36]. CUR also makes H bonds between its side chain B OH and main chain CO of Val- 115 and phenolic OH of Tyr-121. Some minor interactions seen in CUR binding have not been shown. The ligand CUR appears to be stabilized in the active site predominantly by the pi-pi stacking and VDW interactions. These interactions appear to orient the ligand for adequate H-bonding (Figure 2 and Figure 3).

## Conclusion

Curcumin can bind to a number of target molecules to modulate their biological activity. In some instances, such binding has been studied using computational methods like molecular docking. With several target proteins, curcumin has shown strong binding affinity with a binding constant in the nanomolar to micromolar range. In an earlier molecular docking study by the same authors, binding of curcumin to a potential anticancer target enzyme, human stromelysin-1 was detailed [37]. In the present docking study it is seen that curcumin binds to DHFR with an affinity comparable to that of methotrexate, which is an established anticancer drug targeting DHFR. Some of the interactions of curcumin with in the active site of DHFR are similar to those of methotrexate. The flexibility in the structure allows curcumin to bind in a bent conformation in the active site of DHFR thus optimizing interactions on either side of the active site pocket. These docking analyses suggest that curcumin and its derivatives may have similar modes of action as those of known inhibitors of the enzyme like MTX. Curcumin can be considered a potential starting molecule for the design of anticancer drugs targeting DHFR.

## Figures and Tables

**Table 1:** Interaction energies and inhibitor constants (Ki) for the binding of CUR and MTX with DHFRA.

S. No	Parameter	CUR	MTX
1	vdW + Hbond + Desolvation Energy (kcal/mol)	-11.31	-9.98
2	Electrostatic Energy (kcal/mol)	-0.1	-1.49
3	Final Intermolecular Energy (kcal/mol) *	-11.41	-11.47
4	Final Total Internal Energy (kcal/mol)	-1.57	-1.02
5	Torsional Free Energy (kcal/mol)	2.39	2.68
6	Unbound System's Energy (kcal/mol)	-1.57	-1.02
7	Estimated Free Energy of Binding (kcal/mol) **	-9.02	-8.78
8	Estimated Inhibition Constant (298 K), Ki (nM)	243	363
